# Further records of the deep-sea pandalid shrimp *Heterocarpus chani* Li, 2006 (Crustacea, Decapoda, Caridea) from southern India

**DOI:** 10.3897/zookeys.685.13398

**Published:** 2017-07-20

**Authors:** Chien-Hui Yang, Appukuttannair Biju Kumar, Tin-Yam Chan

**Affiliations:** 1 Institute of Marine Biology, National Taiwan Ocean University, Keelung 20224 Taiwan, R.O.C.; 2 Department of Aquatic Biology & Fisheries, University of Kerala, Thiruvananthapuram 695581, Kerala, India; 3 Center of Excellence for the Oceans, National Taiwan Ocean University, Keelung 20224, Taiwan, R.O.C.

**Keywords:** *Heterocarpus chani*, deep-sea, shrimp, India

## Abstract

The commercial deep-sea caridean shrimp *Heterocarpus gibbosus* Spence Bate, 1888 has long been recorded from India and constitutes an important part of the catches in the context of the further development of deep-sea fisheries in India. A recent survey in some deep-sea fishing ports in southern India, however, revealed that all material previously reported as “*H. gibbosus*” is actually a misidentification of its closely related species *H. chani* Li, 2006, which has only recently been reported from India. More detailed comparisons allowed the discovery of more distinctive characters between *H. chani* and *H. gibbosus*.

## Introduction

The commercial deep-sea caridean shrimp *Heterocarpus gibbosus* Spence Bate,1888 was thought to be widely distributed in the Indo-West Pacific (Chace 1985, Crosnier 1988) and in some areas as being rather abundant (Holthuis 1980, Chan and Yu 1987). Recently, however, this species was split into four species (Li 2006, Yang et al. 2010) mainly based on the height of the rostral crest, the development of the boss on the third abdominal somite and the length of the exopod of the third maxilliped. Specimens previously referred to this species from the western Pacific are not the true *H. gibbosus* but are instead *H. abulbus* Yang, Chan & Chu, 2010 and *H. corona* Yang, Chan & Chu, 2010. These two western Pacific species are rather different from *H. gibbosus* in having either a much lower or higher rostral crest (the low crest species, *H. abulbus* also has an indistinct abdominal boss). The other recently described species, *H. chani* Li, 2006, is very similar to *H. gibbosus* but with a much shorter exopod of the third maxilliped (Li 2006, Yang et al. 2010) and is currently mainly known from the South China Sea and the Philippines (Li and Chan 2013) as well as a short report from India (Kuberan et al. 2015). Other than co-occurring together with *H. chani* in the South China Sea and the Philippines, *H. gibbosus* is presumed to be the main species of this species complex in the Indian Ocean.

*Heterocarpus gibbosus* has long been reported from India (e.g., Wood-Mason and Alcock 1892, Alcock 1901, Kemp and Swell 1912, George and Rao 1967, Suseelan 1976, Radhakrishnan et al. 2012, Samuel et al. 2016, see also synonymy list in Fransen 2006) and now forms a major part of the catch in the deep-sea fisheries of India (e.g., Rajan et al. 2001, Kurup et al. 2008, Radhika Rajasree and Madhusoodana Kurup 2011, Rajool Shanis et al. 2012, 2014). However, recently a brief local technical report recorded *H. chani* from southern India (Kuberan et al. 2015). A visit of the third author (TYC) to the deep-sea fishing ports in southern India also found that the abundant materials identified there as “*H. gibbosus*” are actually all *H. chani*. The present work reports this finding. Detailed comparisons of these two species also revealed more differences between them. The material examined is deposited in the Department of Aquatic Biology and Fisheries, University of Kerala (DABFUK), National Taiwan Ocean University (NTOU), Lee Kong Chian Natural History Museum, Singapore (ZRC) and Oxford University Museum of Natural History (OUMNH). Additional materials for comparisons are those reported from the Philippines in Li and Chan (2013) and deposited in NTOU, with 69 specimens of *H. gibbosus* and 66 specimens of *H. chani*. Size measurements given are carapace length (cl) measured dorsally from the postorbital margin to the posterior margin of the carapace. Partial sequences of mitochondrial cytochrome c oxidase I (COI) gene data were generated by following the methods outlined in Yang et al. (2010).

## Taxonomy

### Family Pandalidae Haworth, 1825

#### Genus *Heterocarpus* A. Milne-Edwards, 1881

##### Heterocarpus chani

Taxon classificationAnimaliaDecapodaCaridea

Li, 2006

Figs 1 , 2 , 3

Heterocarpus chani Li, 2006: 362, figs 1–4 (type locality: Philippines).—Yang et al. 2010: 207, fig. 5E.—De Grave and Fransen 2011: 442.—Li and Chan 2013: 133, fig. 1B.—Kuberan et al. 2015: 27, fig. 1.
(?) Heterocarpus gibbosus—George and Rao 1967: 331.—Suseelan 1974: 50, fig. 2-Heterocarpus gibbosus. (*non* Spence Bate, 1888)
(?) Heterocarpus ? gibbosus—Wood-Mason & Alcock, 1892: 368, fig. 6. (*non* Spence Bate, 1888)

###### Material examined.

Sakthikulangara fishing harbor, Kollam district, Kerala, 20 March 2017, 1 ♂ cl 28.65 mm, 2 ovigerous ♀♀ cl 29.9–31.2 mm (NTOU M02049); 1 ♂ cl 33.3 mm, 3 ovigerous ♀♀ cl 26.8–35.8 mm (NTOU M02050), 1 ovigerous ♀ cl 30.0 mm, 1 ♀ cl 24.2 mm (ZRC 2017.0892); 1 ♂ cl 21.3 mm (DABFUK/AR-DEN-30), 1 ovigerous ♀ cl 26.2 mm (DABFUK/AR-DEN-31); 1 ♂ cl 23.0 mm, 1 ovigerous ♀ cl 23.8 mm (OUMNH). Muttom fishing harbor, Tamil Nadu, 21 March 2017, 1 ♀ cl 20.6 mm (NTOU M02051).

**Figure 1.**
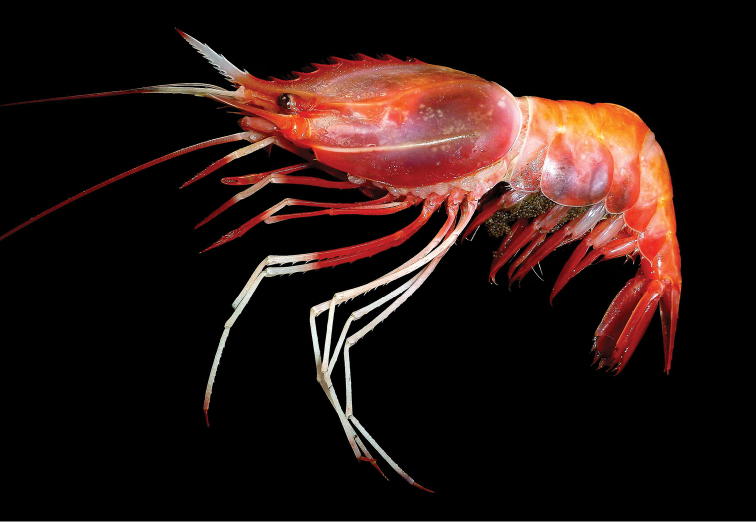
*Heterocarpus chani* Li, 2006, Sakthikulangara fishing harbor, SW India, ovigerous ♀ cl 29.9 mm (NTOU M02049).

###### Diagnosis.

**Figure 2.**
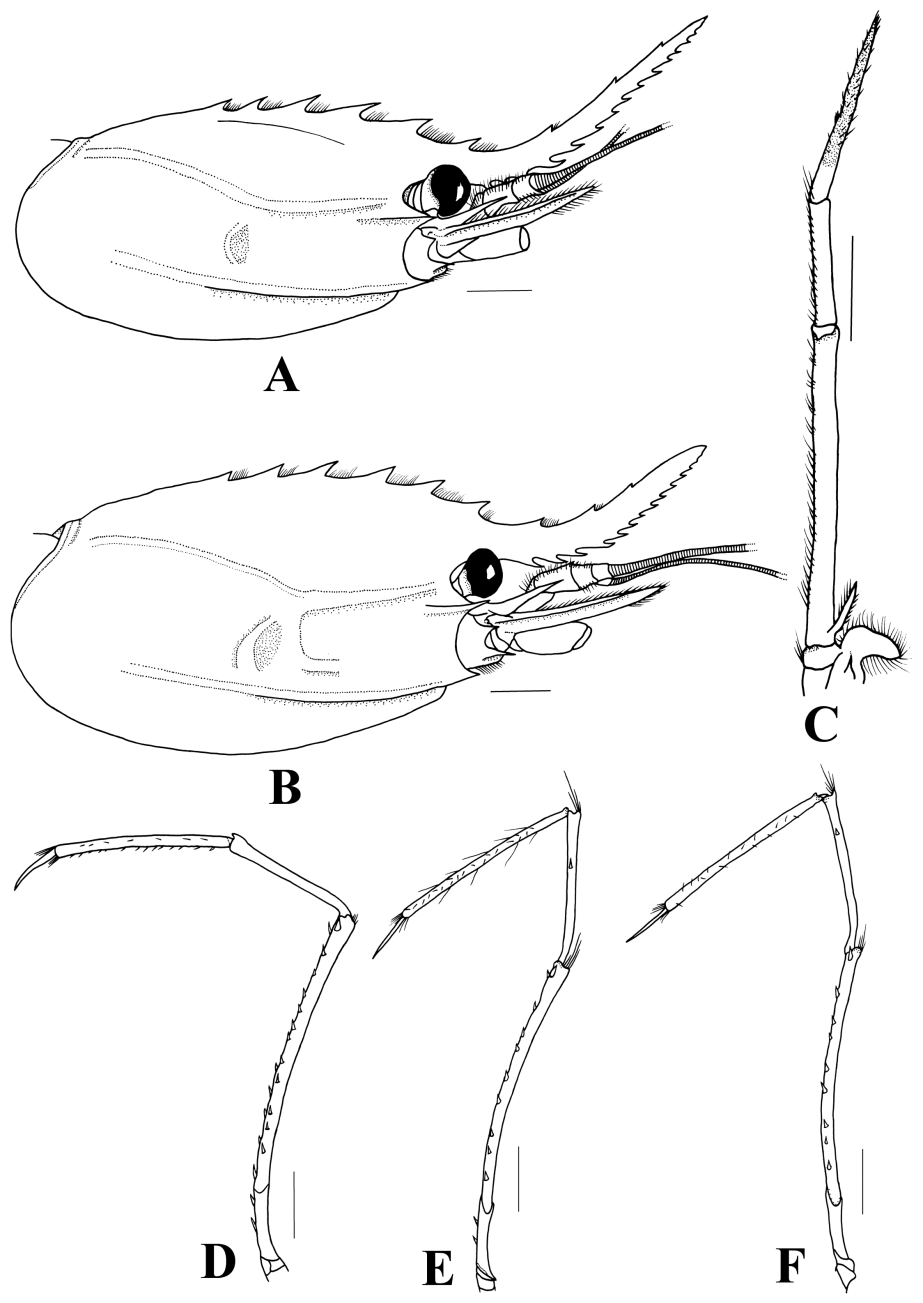
*Heterocarpus chani* Li, 2006, Sakthikulangara fishing harbor, SW India. **A, C–F** ovigerous ♀ cl 26.8 mm (NTOU M02050) **B** ovigerous ♀ cl 31.4 mm (NTOU M02050) **A–B** Carapace, lateral **C** right maxilliped III **D** right pereiopod III **E** right pereiopod IV **F** right pereiopod V. Scales = 5 mm.

**Figure 3.**
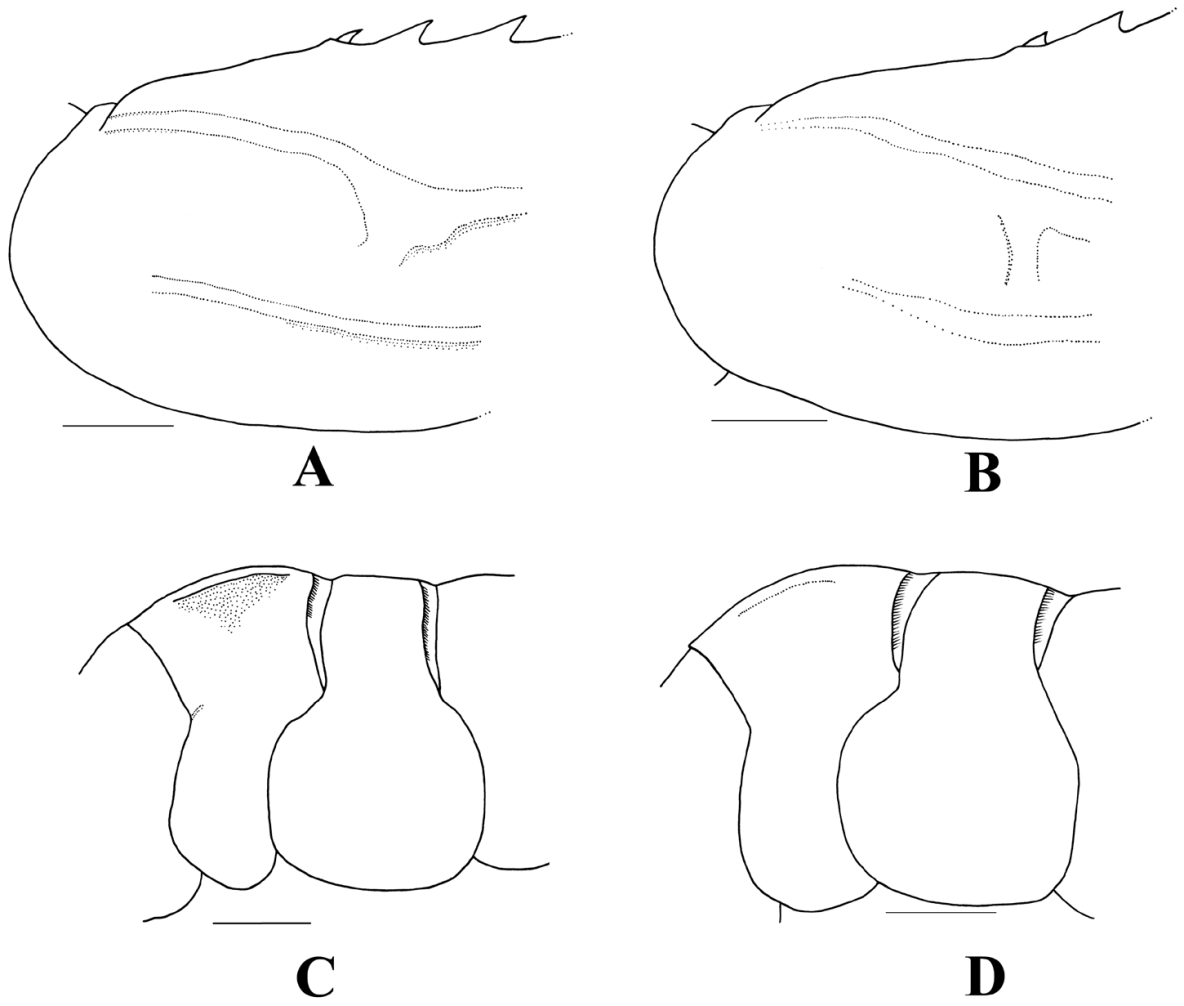
**A, C** *Heterocarpus chani* Li, 2006, Sakthikulangara fishing harbor, SW India, ovigerous ♀ cl 26.8 mm (NTOU M02050) **B, D** *H. gibbosus* Spence Bate, 1888, the Philippines, PANGLAO 2005, stn CP 2359, ♂ cl 25.9 mm (NTOU M00792) **A–B** posterior carapace, lateral **C–D** abdominal somites II and III, lateral. Scales = 5 mm.

Rostrum far overreaching scaphocerite, 0.6–1.0 times as long as carapace. Rostrum dorsally armed with 7–10 teeth including 4–5 teeth on carapace posterior to orbital margin, ventrally armed with 10–16 teeth along entire length but with distal 2–3 teeth obscure. Rostral crest moderately elevated. Two lateral carinae on carapace, postorbital carina extending posteriorly almost to posterior margin of carapace and distinctly recurved downwards at posterior end. Branchiostegal carina sharp and extending posteriorly to 75–80% of carapace length (Figs 2A–B). Abdominal tergites without spine, boss on third somite distinct and with lateral borders somewhat carinate, width 0.2–0.3 and length 0.7–0.8 times as long as somite (Fig. 3C); only pleura IV and V bearing posteroventral tooth. Telson bearing 4 pairs of dorsolateral and 3 pairs of distal spines. Maxilliped III with exopod very short, 0.2–0.3 times as long as antepenultimate segment (Fig. 2C). Pereiopod III with carpus and ischium bearing 0–2 spines, merus with 1–6 mesial and 10–15 lateral spines, dactylus 0.2–0.4 times as long as propodus. Pereiopod IV with carpus bearing 1–2 spines, merus with 1 distinct apical spine and 10–13 lateral spines, ischium with 2 spines. Pereiopod V with carpus bearing 0–1 spines, merus with 10–13 lateral spines, ischium without any spine (Fig. 2D–F).

###### Coloration.

Body generally orange red to rose red, rostrum whitish in anterior half but with tip often reddish. Eyes dark brown. Basal parts of antennular and antennal flagella whitish (more so in former). Scaphocerite with distal part whitish. Ventral lateral carina of carapace sometimes whitish except at tip (i.e. branchiostegal spine). Posterior border of carapace and anterior margin of abdominal somite I whitish. Maxilliped III with penultimate segment and sometimes also distal part of antepenultimate segment whitish. Pereiopod I with posterior part of carpus and sometimes also anterior part of merus whitish. Longer pereiopod II with chela, carpus and anterior part of merus whitish. Shorter pereiopod II only with basal carpus and distal merus whitish. Pereiopods III with propodus, carpus and anterior 1/2-1/3 of merus whitish. Pereiopods IV and V with propodus, carpus, merus and sometimes even entire pereiopod whitish except for reddish dactylus. Eggs greenish brown.

###### Distribution.

Only known with certainty from the South China Sea, Philippines and southern India, at depths of 382 (perhaps as shallow as 200 m, see Kuberan et al. 2015) to 888 m.

###### Remarks.

Although *Heterocarpus gibbosus* Spence Bate, 1888 (type locality: Bohol Sea, The Philippines, see Li et al. 2007, Anonymous 2009) has long been reported from India, going back to soon after the original description of the species (Wood-Mason and Alcock 1892). Although recently considered as an important catch in the local deep-sea fishery (e.g., Rajan et al. 2001, Kurup et al. 2008, Rajasree and Kurup 2011, Rajool Shanis et al. 2012, 2014), the abundant specimens observed in three deep-sea fishing ports in southern India (Sakthikulangara, Muttom and Tuticorin, only specimens kept as vouchers were listed in Material examined) all actually represent *H. chani* without exception. Thus, the brief record of *H. chani* in India by Kuberan et al. (2015) is confirmed. The southern Indian material of *H. chani* agrees well with that reported from the South China Sea and the Philippines (Li 2006, Li and Chan 2013). The only observed difference is the length of the rostrum being more frequently shorter in the Indian population. About half of the Indian specimens have the rostrum 0.6–0.7 times as long as the carapace in contrast to only about 1/10 of the comparative Philippines material with a shorter rostrum. Comparison of the COI sequences (657 bps) between the short (GenBank accession nos. MF149971-MF149973) and long rostral (MF149974) forms in the Indian material shows only 0.2–0.6% divergence, while genetic divergences between the Indian and Philippine materials (GQ302748, GQ302750, GQ302752, GQ302754) are 2.1–2.7 %. COI sequence divergences of less than 3% are generally considered to be intraspecific in decapod crustaceans (e.g. Darling 2011, Vergamini et al. 2011, Yang et al. 2016). The coloration of the Indian material also generally agrees with that from the Philippines (Fig. 1, Li 2006: fig. 4, Yang et al. 2010: fig.5E, Li and Chan 2013: fig. 1B).

While *H. chani* exhibits a high genetic divergence from *H. gibbosus* (GQ302740, GQ302742, GQ302744, GQ302746, with 10.0–11.5% COI sequence divergence, also see Yang et al. 2010), the two species are morphologically very similar and mainly differ in the relative length of the exopod on the third maxilliped (Li 2006). More careful comparison of the present material reveals that there are three more, somewhat, subtle differences between these two species. The posterior end of the postorbital carina is distinctly ridged and recurved downwards in *H. chani* (Fig. 3A), but becomes rather indistinct and not bending downwards in *H. gibbosus* (Fig. 3B). The abdominal boss is more distinct and with the lateral borders ridged in *H. chani* (Fig. 3C) whilst in *H. gibbosus*, the abdominal boss is relatively less distinct and with the lateral borders not forming ridges (Fig. 3D). The posterior pereiopods each have a distinct red band on the anterior part of the merus in *H. gibbosus* (Li 2006: fig. 6, Yang et al. 2010: fig. 5B, Li and Chan 2013: fig. 1C), while such red bands are lacking in *H. chani* (Fig. 1, Li 2006: fig. 4, Yang et al. 2010: fig. 5E, Li and Chan 2013: fig. 1B; Kuberan et al. 2015: fig. 1).

Although more differences are now enumerated between *H. chani* and *H. gibbosus*, it still cannot be deduced if the numerous reports of *H. gibbosus* from India in reality refer to that species or indeed solely represent *H. chani*, because all these records are too brief or did not list nor discuss any of the above distinctive characters. However, the “*H. gibbosus*” specimens from SW Cochin reported by George and Rao (1967) were described as having an abdominal boss with rather prominent median carination, and therefore likely represent *H. chani* instead. The “Heterocarpus ? gibbosus” illustrated by Wood-Mason and Alcock (1892: fig. 6), though lacking an abdominal boss, likely also represents *H. chani* since it has a shorter rostrum and with the posterior end of the postorbital carina sloping downwards. The rough line-drawing of “*Heterocarpus gibbosus*” provided in Suseelan (1974: fig. 2) also shows a posteriorly recurved downward postorbital carina. Thus, whether *H. gibbosus* truly occurs in India continues to be in need of confirmation.

## Supplementary Material

XML Treatment for Heterocarpus chani
